# Devolution of power, revolution in public health?

**DOI:** 10.1093/pubmed/fdw031

**Published:** 2016-05-10

**Authors:** Srinivasa Vittal Katikireddi, Katherine E. Smith, David Stuckler, Martin McKee

**Affiliations:** 1 Public Health, MRC/CSO Social and Public Health Sciences Unit, University of Glasgow, Glasgow G2 3QB, UK; 2 Global Public Health Unit, School of Social and Political Sciences, University of Edinburgh, Edinburgh EH8 9LD, UK; 3 Political Economy and Sociology, Department of Sociology, University of Oxford, Oxford OX1 3UQ, UK; 4 European Public Health, European Observatory on Health Systems and Policies, London School of Hygiene and Tropical Medicine, London WC1H 9SH, UK

## Introduction

On 8 May 2015, the Conservative party won an unexpected majority in the UK general election.^1^ Commentators argued that a potentially decisive factor was concern in England about the Scottish National Party (SNP) forcing a minority Labour government to ‘dance to the SNP's tune.’^2^ Meanwhile the SNP won a historic number of seats in Scotland, capturing 56 of the 59 Westminster constituencies.

The election took place against a backdrop of debate about the future of the UK. Although the Scottish independence referendum was lost, it was widely agreed that the status quo no longer remained feasible. At the same time, there is pressure to revisit Welsh devolution^3^ and the Westminster government has proposed devolving certain powers to locally elected decision-makers in English regions.^4^ Meanwhile, the new Conservative government has committed to a referendum on continuing European Union membership, reflecting calls for ‘Brexit’.^5^ Although initially emanating from those on the right of the political spectrum, there is now some support from the left,^6^ making it a real possibility and likely to trigger a second Scottish referendum. One way or another, substantial political change is looming.

If we conceive of ‘health’ as ‘health care’ (as many political scientists do), it could be argued that further devolution has little significance since responsibility for the NHS is already devolved to each national administration.^7^ Yet, most of the major determinants of population health and health inequalities lie outside the health system, often in areas for which responsibility has so far remained in Westminster.^8,9^ For example, macro-economic and welfare policies have large health impacts, potentially more so than health services and,^10^ while the relatively modest scale of devolution has not yet led to marked divergence in such policies, this looks set to change. We apply a political science framework focussing on ideas, interests and institutions to demonstrate potential opportunities and threats to public health arising from political change, so that researchers and practitioners can better engage with the political determinants of health.

## Political devolution in the UK

In contrast to federal systems (where power is shared between regions and the nation-state), the UK was, until the late 20th century, characterized as having a strong central government—the so-called ‘Westminster Model’.^11^ In 1999, the Labour government began a period of rapid constitutional change. Initially, it created new centres of power, including the Scottish Parliament, Welsh Assembly, Northern Ireland Executive and Greater London Authority.^12,13^ These institutions were not created equal—each gained different political portfolios reflecting pre-existing differences among them. The Scottish Office, for example, had longstanding responsibility for adapting Westminster legislation to Scotland's separate legal and educational systems. The creation of a modern Scottish Parliament went beyond this, assuming legislative responsibility in devolved policy areas such as health, education and justice.^14^ The Welsh Assembly, in contrast, had less of a foundation for operating independently to Westminster and only achieved limited legislative powers in 2006. Meanwhile, the situation in Northern Ireland has been complicated by devolution sitting within the framework of an international agreement with the Republic of Ireland,^15^ and has been interrupted by breakdowns in the Northern Ireland peace process. For all three regions, health policy was one of the most important policy areas initially devolved. The Greater London Authority has no legislative or executive responsibility for health but is responsible for policies such as transport that impact on health.

The relationship between the devolved administrations and the UK Parliament is complicated by two issues. First, as devolution did not lead to a federal UK structure since an English elected assembly/parliament was not created, there have been concerns about the equity of a system allowing Scottish and Welsh MPs to vote on matters which may only affect communities living in England (the so-called ‘West Lothian’ question, discussed below). Second, only some elements of a policy area may be ‘devolved’^14^ meaning responsibility for decision-making is not always clear, with jurisdiction potentially stretching across devolved institutions, the UK Parliament and European institutions. Additionally, as the SNP has noted, the policy links across the UK mean decisions about England may have considerable implications for other territories.^16^ Adding to this complexity, devolution is better understood as a ‘process’ evolving over time, rather than an ‘event’.^14^

## The UK: a process of growing fragmentation

Much of the momentum for constitutional reform derives from the recent Scottish referendum, intended to settle the question of Scottish independence for ‘a generation’.^17^ Two days before the vote, facing possible defeat, the leaders of the three main UK political parties very publicly vowed that ‘The Scottish Parliament is permanent and extensive new powers for the Parliament will be delivered’.^18^ Lord Smith, an independent cross-bench peer, was therefore asked to produce recommendations for devolving further powers. The Smith Commission's report included measures to allow the Scottish Parliament to control aspects of welfare policy (including benefits for carers and disabled people), support for unemployed people and aspects of taxation (including a proportion of income tax and value added tax), all of which have impact health.^19^ In May 2015, the Scotland Act was introduced to the House of Commons and, at the time of writing, is proceeding through the legislative process.^20^

However, power will not only flow from Westminster to Scotland. Once the Scottish referendum votes were in, it became clear that any new powers would be part of a broader package of UK-wide change, giving greater powers to English cities and threatening to diminish the rights of Scotland-based members of the UK (Westminster) parliament. On the morning of the Scottish referendum result, David Cameron surprised many by saying:

We have heard the voice of Scotland - and now the millions of voices of England must also be heard. The question of English votes for English laws - the so-called West Lothian question - requires a decisive answer.”^21^

English votes for English laws (EVEL) has been presented as a solution to the West Lothian question (named after the constituency of Tam Dalyell, the MP who first raised the issue), which asks why Scottish MPs can vote at Westminster on matters not affecting their constituents.^14^ For example, New Labour's establishment of Foundation Trusts in 2003 was only possible because Scottish Labour MPs supported the policy, which did not affect the Scottish NHS.^22^ EVEL could bring about a profound change in the power of different political parties. Based on recent voting patterns, this seems likely to favour the Conservative Party. The government's proposals,^23,24^ by means of a change to the standing orders of the House of Commons, would have avoided the difficulties of gaining consent from the House of Lords. While it was withdrawn in the face of opposition from the Conservative backbenches, EVEL remains a government goal.

George Osborne, Chancellor of the Exchequer, has called for the imbalance across the English regions to be addressed, seeking to ‘make the cities of the north a powerhouse for our economy again – with new transport and science and powerful city governance’.^25^ The promise of greater powers is being rapidly implemented, with new democratic posts introduced and existing institutions strengthened, albeit with many questions still unanswered.^4,26^ Greater Manchester is at the forefront, with a newly established mayor (responsible for a devolved transport and housing budget, as well as influence over strategic planning). The Greater Manchester Combined Authority's influence will also increase so it jointly commissions health and social care (with local clinical commissioning groups) and controls some aspects of return-to-work initiatives, children's services and business development,^27,28^ although how this can be reconciled with the Health and Social Care Act remains unexplained^29^ and there are already signs of tensions emerging.^30^ While greater political engagement appears to have followed Scottish devolution, it remains uncertain whether the same will happen in England—especially in Manchester, where the local electorate voted against introducing a Mayor.^31^

Increased decision-making powers have been transferred to other devolved institutions too. The Commission on Devolution in Wales was established to review the Welsh Assembly's powers and published its recommendations in two parts.^32^ The first part called for the Welsh Assembly to have ownership over some aspects of fiscal policy (such as the ability to vary income tax and responsibility over stamp duty) and was implemented in legislation through the Wales Act 2014. The second set of proposals included decision-making over police and youth justice and these are being implemented incrementally, with a Wales Bill expected shortly.^3,33^ Northern Ireland, in contrast, has had little change in its decision-making powers, partly as a result of the Assembly's suspension between 2002 and 2007 following the ‘troubles’. While no formal review over powers has occurred, civil society is debating the issue.^34^

## Understanding policy change: ideas, institutions and interests

Within political science and policy studies, three recurring concepts are often employed to explain policy change: ideas, institutions and interests, sometimes referred to as the ‘3-Is’ framework.^35,36^ Each is now considered, with a view to identifying questions for public health professionals to consider—summarized in Table 1.

**Table 1:** Questions to ask when developing a public health advocacy strategy in an era of devolution

*Issue*	*Description*
Ideas	What public health problems are priorities and might the changing political circumstances increase/reduce opportunities to promote these priorities? What are the optimal policy solutions to these issues, from a public health perspective, and how does the local political climate help or hinder their adoption?
Institutions	Which political institutions are relevant and what powers do they have to tackle public health policy priorities?
Interests	Who are the different stakeholders with an interest in influencing decisions that impact on public health and how will changing political circumstances enable (or constrain) these different interests from influencing policy?

### Ideas

In seeking to understand what explains policy change, many political scientists focus on the role of ideas.^37^ Smith argues that there are distinct idea-types, each with characteristics that aid, or restrict, their potential to influence policy.^38^ Taking the example of minimum unit pricing for alcohol, we suggest that devolution may play help stimulate new policy ideas.^38^ The exceptionally high burden of alcohol-related harm in Scotland^39^ regularly attracts negative media and international policy attention.^40,41^ While there is compelling evidence on the effectiveness of price-related interventions,^42^ the Scottish Government did not have responsibility for alcohol taxation and was therefore unable to increase alcohol's price through traditional policy measures.^43^ In a political climate more accepting of government intervention than Westminster, an opportunity was available for alternative ways of tackling alcohol-related harms and ‘minimum unit pricing’ (which introduces a floor price, based on alcohol content, below which alcohol cannot be sold) was proposed.^44,45^ Crucially, the evidence suggests it will yield greater health benefits than equivalent taxation changes.^46^ Consequently, the limited legislative powers of devolved institutions (discussed further below) may drive innovation resulting in more effective policy. However, smaller jurisdictions may lack capacity to develop and take forward new ideas.^47^

Devolution of further powers to Scotland and Wales alters the political context within which a broader range of decisions are made. Looking forward in Scotland, the SNP has consistently opposed austerity policies,^48^ so devolution of welfare policy presents an opportunity for alternative ideas about welfare to move onto the Scottish policy agenda. NHS Health Scotland, a Special Health Board that provides the Scottish Government with advice, is already arguing for a different approach.^49^ If policy diverges between Scotland and England, there will be opportunities for researchers to contrast the health effects of these two differing approaches. While these ‘natural experiments’ could be invaluable in research terms,^50,51^ policy variations could exacerbate geographic inequalities across the UK, with implications for front line health staff.

### Institutions

Context, including the history and organization of political institutions, also shapes decision-making.^52^ As Immergut puts it, ‘institutions—be they the formal rules of political arenas, channels of communication, language codes or the logics of strategic situations—act as filters that selectively favour particular interpretations either of the goals toward which political actors strive or of the best means to achieve these ends.’^53^ Under current arrangements, only the UK government has responsibility for policy issues such as defence, foreign affairs and macro-economic policy. Policy proposals to improve population health must therefore compete for legislative time against myriad issues at Westminster. In contrast, devolved assemblies have concentrated on domestic issues, such as health, education, and social care.^14^ In some cases, where issues cut across the policy responsibilities of multiple institutions/levels, this division may limit the potential for holistic policy responses. On the other hand, the smaller size of devolved institutions may facilitate joined-up thinking—this has been the intention of the Scottish Government doing away with departmental divisions to promote a ‘whole-of-government’ approach to complex social issues,^54^ although its success has yet to be evaluated. In addition to party political variation, institutional differences could help with the sub-national introduction of health-enhancing policies currently low on the UK political agenda^55^—a feature observed elsewhere in Europe (Box 1).

Box 1 **Federalism in other parts of Europe**

Regions in federal countries have often been able to pursue public health policies that have been unachievable at national level. In Germany, for example, the tobacco industry has long been extremely powerful.^56^ The national government introduced a ban on smoking in federal buildings and public transport in January 2007 but was unable to legislate for other public places. More ambitiously, the regions of Baden-Württemberg and Lower Saxony banned smoking in restaurants, bars and clubs in August 2007, followed in October by Hesse. However, other regions took longer and, in many, the bans that emerged were limited.

Also in Germany, North Rhine Westphalia developed innovative methods of establishing health targets, bringing together the normally fragmented public health and healthcare actors, as did Catalonia, in Spain.^57^ Elsewhere in Spain, the Basque Country has been a pioneer in the development of models of integrated care. In Sweden, the Västra Götaland region developed an Action Plan for Health Equity that has attracted considerable attention in other parts of the country.

If these trends continue, public health may benefit. On the other hand, increasing fragmentation of power across multiple political institutions may create new barriers to tackling ‘wicked issues’, which require coordinated action across different sectors and levels of government. Devolution already means responsibility for tackling cross-cutting policy areas within the UK can be unclear^12^ and the problems are especially acute where action is required at a European or international level. The English Department of Health also takes lead responsibility for the entire UK (and dependent territories) in international fora, with little scope for their distinctive voices to be heard.

While opportunities for promoting public health considerations may increase following a proliferation of political fora, the capacity of each decision-making institution to coordinate action across sectors may be limited. Opportunities may arise in different parts of the country at different times, but coordination across sectors (such as health, welfare and taxation policy) could become more difficult in this context, requiring alignment across all levels of government, rather than just at Westminster. There is also a risk that policy areas perceived as politically unpopular (such as reforming taxation) may be neglected by institutions, despite increased powers, to avoid assuming political responsibility. Box 2 illustrates some of these challenges in relation to health inequalities policy.

Box 2 **Health inequalities policy in the devolved UK**

Efforts to reduce health inequalities provide an example of the difficulties that may arise as a consequence of the lack of a single governing institution. Such inequalities are commonly viewed as a ‘wicked problem’ that requires coordinated actions across multiple policy sectors (such as employment, welfare, health, education). However, coordinating actions across all these sectors may become more difficult in any one of the UK territories or regions if the number of political venues increases. A recent survey of 92 health inequalities researchers working in the UK found that the majority believed that the most effective policy proposals for reducing health inequalities involved more progressive taxation, achieving a minimum income and providing better (and more targeted) public services and support.^58^ The top five proposals, summarized below, all involve policy decisions which are currently shaped by devolved, UK and European policies:

1. Review and implement more progressive systems of taxation, benefits, pensions and tax credits that provide greater support for people at the lower end of the social gradient and do more to reduce inequalities in wealth.

2. Develop and implement a minimum income for healthy living.

3. Increase the proportion of overall government expenditure allocated to the early years and ensure this expenditure is focussed progressively across the social gradient.

4. Increase social protection for those on the lowest incomes and provide more flexible income and welfare support for those moving in and out of work.

5. Support an enhanced home building program and invest in decent social housing to bring down housing costs.

Following the Smith Commission's report, Scotland's responsibility for many of these areas remains unclear and their introduction in a coordinated manner is likely to be difficult. For example, increased expenditure on social protection by the Scottish Government may only be achievable if it also has direct control over revenue accruing from any tax-varying decisions. Furthermore, the lack of clarity around where responsibility lies for these policy areas may provide a means for politicians to avoid taking responsibility for these potentially politically sensitive topics.

Political institutions within the UK are also subject to constraints imposed by supranational organizations and agreements (e.g. European Union policy and international trade agreements). Historically, many of these evolved from negotiations to foster ‘free trade’, that is to help increase trade between countries by removing potential barriers to the free movement of goods and services.^59,60^ A primary focus on trade may result in policies that harm other important policy areas (such as health or environmental concerns).^61^ Over time, many institutions (and especially the European Union) have broadened their scope to include human rights, monetary policy as well as health, thus implying the dominance of economic interests may have lessened.^59^ The limited empirical evidence is currently somewhat contradictory, suggesting that within the European Union, trade interests remain influential but by no means universally dominant.^62,63^ Meanwhile, the increased economic responsibility of regional authorities in England provides an opportunity for the public health voice to argue for economic development that benefits population health and meets the needs of the most disadvantaged.

The rules and cultural norms through which policy is made influences both whose voices are heard and also the type of evidence sought.^64^ As political reforms are implemented, the processes through which policy communities debate and make decisions changes.^65,66^ This therefore provides an opportunity to shape the processes by which decisions are made, not just the content of specific decisions. This presents public health professionals with an opportunity to encourage a culture in which health concerns are embedded in long-term public policy debates.

### Interests

Several political science theories highlight how policy is strongly influenced by a diverse range of interests, including public health advocates, corporate interests and civil society.^52^ Changes in political institutions present new opportunities for such interests to influence policy and could lead to existing policy networks being reshaped or new ones emerging.^67^ This could serve to strengthen public health advocates, as their voices may be heard more readily—perhaps especially when sub-national political parties campaigning for independence are seeking to develop distinctive policy approaches.^43,68^ In the UK case, sub-national leadership has been important in developing effective tobacco control.^69^ On the other hand, more dispersed political power may overwhelm the small community engaged in public health advocacy. Large corporate interests face fewer resource constraints and may, therefore, be relatively advantaged. For example, decisions to withhold licences for alcohol sales in a heavily provided area of Edinburgh were overturned following appeals made by a team of lawyers on behalf of Sainsbury's supermarket.^70^ Countering a shift in power toward vested interests will require close collaboration amongst international alliances of public health advocates.

Vested corporate interests could benefit from the overlapping remits of different political institutions mentioned above by choosing to frame the policy debate in ways that advantage them. For example, the same policy problem can often be presented as primarily a health or trade issue.^44,71–73^ This is illustrated by experience with tobacco control following devolution. The Scottish tobacco display ban, introduced as a public health measure, has faced numerous unsuccessful challenges in the Scottish, UK and European courts, primarily on account of its alleged trade impact.^74^ As the number of policymaking ‘levels’ increases, so too does the scope for vested interests to re-locate policy issues to the jurisdictions they believe are most likely to favour them. It also provides them with an opportunity to mount legal challenges, based on claims that particular measures exceed the powers of the subordinate legislature.^75^ Although rarely successful over the long-term, these challenges can introduce considerable delay and distract public health officials from making progress elsewhere.

## The need for a politically responsive public health

Further change to the UK's political institutions and accompanying policy divergence across the UK's countries and regions seem inevitable. These reforms, coupled with a potential UK exit from the European Union, will bring both opportunities and challenges for public health. If the public health community is serious about embedding ‘health in all policies’ over the long-term, we will need to ensure our voice is clearly heard at all levels of decision-making and provide evidence that is tailored to evolving institutional responsibilities in a changing political climate. Looking forward, there appears to be some political appetite for divergence in welfare, housing and potentially even fiscal policy, providing greater opportunities for evaluating natural policy experiments relating to key social determinants. Public health professionals should be at the forefront in developing the health-focussed evidence-base for these areas, ready to advocate for the health needs of their populations when opportunities present themselves.

## Funding

S.V.K. was funded by the Chief Scientist Office of the Scottish Government (MC_UU_12017/4). DS is funded by ERC HRES 313590 and a Wellcome Trust Investigator Award.

## Conflicts of interest

S.V.K. has previously been seconded to Scottish Government during his public health training. The authors declare they have no other conflicts of interest.
