# Devolution of power, revolution in public health?

**DOI:** 10.1093/pubmed/fdx086

**Published:** 2017-08-18

**Authors:** Srinivasa Vittal Katikireddi, Katherine E Smith, David Stuckler, Martin McKee

*J Public Health (Oxf)* 2017; 39 (2): 241–247. doi: 10.1093/pubmed/fdw031

The funding information should have included the following statement:

DS is funded by ERC HRES 313590 and a Wellcome Trust Investigator Award.

The online article has been corrected.

